# Factors associated with length of stay and death in tube‐fed patients: A cross‐sectional multicentre study

**DOI:** 10.1002/nop2.774

**Published:** 2021-01-27

**Authors:** Leticia Alves Freitas, Alex Luís Fagundes, Patrícia Rezende do Prado, Marta Cristiane Alves Pereira, Adriane Pinto de Medeiros, Ligia Menezes de Freitas, Thalyta Cardoso Alux Teixeira, Janine Koepp, Rhanna Emanuela Fontenele Lima de Carvalho, Fernanda Raphael Escobar Gimenes

**Affiliations:** ^1^ University of São Paulo at Ribeirão Preto College of Nursing São Paulo Brazil; ^2^ Federal University of Acre Acre Brazil; ^3^ Faculty of Science and Technology of Campos Gerais Minas Gerais Brazil; ^4^ Paulista University São Paulo Brazil; ^5^ University of Santa Cruz do Sul Rio Grande do Sul Brazil; ^6^ Ceará State University Ceará Brazil

**Keywords:** enteral nutrition, length of stay, mortality, nursing care, patient safety, quality of health care

## Abstract

**Objectives:**

To analyse the factors associated with length of stay (LOS) and death in nasogastric/nasoenteric tube (NG/NET)‐fed patients.

**Design:**

A cross‐sectional multicentre study.

**Method:**

Data collection took place from October 2017–April 2019, and the sample consisted of 365 participants from seven Brazilian hospitals. Demographic, clinical and therapeutic data were collected from the patients’ medical records. Data analysis was performed using bivariate and multivariate tests, considering a significance level of *p*<.05.

**Results:**

Most patients were male, older adults, with high risk of death and highly dependent on nursing care. The LOS was associated with age, patient care complexity and length of NG/NET use. Death was associated with patient age. In the multivariate analysis, patients highly dependent on nursing care, and intensive and semi‐intensive care had a greater chance of dying when compared with patients receiving minimal care. Screening for factors affecting LOS and death is important to plan effective nursing care.

## 1 INTRODUCTION

Severely ill patients usually present different degrees of inflammation, metabolic stress, comorbidities and haemodynamic instability that can result in reduced calorie and protein intake and increased energy expenditure. There are several reasons why oral ingestion may not be possible in these patients, such as dysphagia secondary to stroke, neuromuscular disease, Parkinson's disease, altered level of consciousness, mechanical ventilation and psychological and/or psychiatric factors, such as anorexia nervosa (Scott & Bowling, 2015). Accordingly, patients who are unable to meet their nutritional requirements may benefit from the use of enteral nutrition (Idrissi et al., 2015; Ojo et al., 2019; Scott & Bowling, 2015; Singer et al., 2019).

Enteral nutrition is administered through an enteral access device, distal to the oral cavity, through which the patient receives nutrients directly into the gastrointestinal tract (Boullata et al., 2017). An enteral access device is defined as a tube, catheter or stoma, positioned through the nostrils, mouth, stomach or small intestine directly into the gastrointestinal tract (Lord, 2018; Lewis et al., 2018), used to make enteral nutrition possible, as well as for the administration of fluids and medications, gastric drainage and decompression (Boullata et al., 2017).

Among all enteral access devices, the nasogastric/nasoenteric tube (NG/NET) is the most used in the hospital environment for temporary enteral nutrition support, of up to −6 weeks (Boullata et al., 2017; Lord, 2018). This short‐term enteral access device can be inserted at the bedside by trained nurses and is easily removed when no longer needed (Lord, 2018). The NG/NET provides nutritional support for patients with a functional gastrointestinal tract to promote nutritional status, improve wound healing and enhance the patients’ quality of life (Ojo et al., 2019).

Although common in hospital settings (Best & Hitchings, 2010; Ojo, 2015), NG/NET is associated with serious and fatal adverse events that result in poor patient outcomes, increased length of stay (LOS) and death (Awad et al., 2017; Filho, 2018; Motta et al., 2021). Furthermore, the degree of complexity, number of comorbidities and age are factors that contribute to NG/NET‐related adverse events and in‐hospital mortality (Blumenstein et al., 2014; Mundi et al., 2018; Soares et al., 2015).

Several studies have shown the association between long‐term enteral access devices and early mortality (Johnston et al., 2008; Miranda et al., 2019; Pih et al., 2018) in critically ill patients (Vest et al., 2018). In addition, the association between LOS and enterally tube‐fed critically ill patients has also been reported (Anziliero & Beghetto, 2018; Atasever et al., 2018; Ortega et al., 2017). However, studies involving NG/NET‐fed adult patients from clinical wards are still incipient, especially in low‐ and middle‐income countries (Gimenes et al., 2019).

The investigation of this relationship is important because LOS and death are determinant of cost and are commonly used care quality measures (Awad et al., 2017). Therefore, this understanding may help nurses to develop strategies to reduce the risks and improve patient outcomes. The purpose of the study was to analyse the factors associated with LOS and death in tube‐fed patients.

## 2 METHOD

### 2.1 Study design

This study was part of a broader research project on feeding tube‐related adverse events (Gimenes et al., 2019). This was a cross‐sectional multicentre study.

### 2.2 Setting and participants

The study included adult patients admitted to the medical wards of seven Brazilian hospitals (a mix of community and university, public and private hospitals). The inclusion criteria were patients over 18 years of age; who were admitted to a medical ward with an NG/NET or patients who required the insertion of an NG/NET during hospitalization; and inpatient stay of at least 24 hr. Only the first admission of patients meeting the above inclusion criteria that were readmitted during the study period was considered.

Sample size was determined by stratified random sampling with proportional allocation by strata, where each stratum was formed by the units of each hospital. Adopting the parameters of relative error of 20%, level of significance of 5% and the total population of 4,573 patients with an NG/NET in a period of six months, a total sample size of 281 patients was calculated (Gimenes et al., 2019). However, the observations performed exceeded the required sample size, totalling 365 patients.

### 2.3 Instruments

The data collection instruments consisted of electronic forms developed by the research team and evaluated, in terms of face and content validity, by a panel of five experts. The forms were developed in the Portuguese language using the SurveyMonkey^®^ online platform. The experts were selected through the analysis of their curricula stored in the Brazilian National Council for Scientific and Technological Development database and were invited to participate in the study. On acceptance, the access links to the electronic forms were made available to the experts for evaluation. The modified electronic forms were tested through a pilot study with five patients admitted to the medical wards, from the first day of use of the NG/NET to discharge from the ward (Gimenes et al., 2019).

### 2.4 Data collection

Data collection took place from October 2017–April 2019. In each hospital participating in the study, a regional coordinator was appointed to ensure the quality and comprehensiveness of the data collected. For the data collection, a mobile device (cell phone or tablet) was used, from the first day of use of the NG/NET on the ward (or from the first day of hospitalization if the patient was admitted to the ward with the tube) until discharge from the ward (due to death or non‐death). Demographic, clinical and therapeutic data were collected from the patients’ medical record on admission and included date of admission to the unit/ward; date of birth; city and state of origin; sex; race; marital status; and education level.

The clinical variables were as follows: main medical diagnosis, according to the International Classification of Diseases, 10th edition; Charlson Comorbidity Index (CCI) adjusted for age (Charlson et al., 1987); final score of the Patient Classification System (Fugulin et al., 2005); and level of consciousness, assessed through the ACDU Scale (alert, confused, drowsy and unconscious) (McNarry & Goldhill, 2004). The therapeutic variables were as follows: date and main reason for NG/NET removal; date and time of patient discharge; reason for discharge (death or non‐death); and data related to the NG/NET, such as distal tip position and tests performed to confirm tube placement.

In this study, disease severity was assessed using the age‐adjusted CCI (Charlson et al., 1987). The CCI is an index that measures the severity of the patient, regardless of the main diagnosis, and is able to predict the one‐year mortality of patients. The final score is the result of the sum of the weights assigned to the comorbidities recorded as secondary diagnoses; the higher the score, the greater the risk of the patient dying. According to the final score, the risk of death can be classified as low (score 1–2), moderate (score 3–4) and high (score ≥ 5) risk. Comorbidities should be adjusted for confounding variables, including age. The age‐adjusted CCI allows quantification of the prognostic effect of age and comorbidities on the risk of death (Tian et al., 2017; Yang et al., 2017).

The Patient Classification System proposed by Fugulin et al. (2005) was used to access patient care complexity. The system includes scores that vary from 1–4 (1 = the best degree and 4 = the worst degree) for nine critical indicators: mental status, oxygenation, vital signs, mobility, ambulation, feeding, body care, elimination and therapy. The classification of the final score is given as minimum (score from 9–14), intermediate (score from 15–20), high‐dependency (score from 21–26), semi‐intensive (score from 27–31) and intensive (score greater than 31) care.

The level of consciousness was assessed using the ACDU Scale (McNarry & Goldhill, 2004). It is a simple, quick and useful scale to assess, at the bedside, the patient's level of consciousness. It is often used by nurses and other healthcare providers in different contexts. This scale has also proved to be superior to the alternatives in the early identification of neurological deterioration in critically ill patients on wards.

### 2.5 Data analysis

Data were analysed using the R software, version 3.5.3. Descriptive statistical analysis included absolute and relative frequencies, to characterize the tube‐fed patients and to describe the clinical and therapeutic variables. To analyse the association between the LOS response variable and the explanatory variables (patient care complexity, disease severity and level of consciousness), the Jonckheere–Terpstra test was used. Crosses were also performed between the LOS variable and the age and length of NG/NET use explanatory variables. In this case, Spearman's correlation coefficient was applied.

To analyse the association between death and the explanatory variables (patient care complexity, disease severity and level of consciousness), the Cochran–Armitage chi‐square test was used. To analyse the association between death and the variables age and time of NG/NET use, the Mann–Whitney test was applied.

A binary regression model was run from factors associated with LOS and death in tube‐fed patients adjusting for age, disease severity (measured by the age‐adjusted CCI), patient care complexity (measured by the Patient Classification System), level of consciousness (according to the ACDU scale) and length of NG/NET use (in days).

Finally, to perform the logistic regression analysis, the response variables were LOS (in days) and death (yes/no). The selection of explanatory variables for the final model was performed using the likelihood ratio test. All analyses were performed considering a 5% significance level (α = 0.05).

### 2.6 Ethical considerations

According to resolution 466/2012 of the National Health Council, which addresses ethics in research with human subjects, informed consent was obtained from each patient, or their guardian, prior to inclusion in the study. The multicentre study was approved by the Research Ethics Committee. The Strengthening the Reporting of Observational Studies in Epidemiology (STROBE) Statement checklist was used to guide the construction of this article (File S1).

## 3 RESULTS

The study participants presented a mean age of 64 years (66.54; *SD*: 16.9); most were male (192; 52.6%), white (240; 65.8%), married (161; 44.1%), with elementary education (131; 35.9%) and residents of the state of São Paulo (231; 63.3%). Regarding the period of admission to the ward, 49.6% (*N* = 181) were admitted during the afternoon shift, with just over half using NG/NET at the time of admission (214; 58.6%). Most patients were admitted to the general wards (144; 39.5%), followed by the oncology (51; 14.0%) and neurology (33; 9.0%) specialities.

The main diagnosis was concentrated in the neoplasm (tumour) chapter of the *International Classification of Diseases, 10th* *edition* (55; 18.3%), followed by diseases of the circulatory system (46; 15.3%) and diseases of the nervous system (32; 10.6%). In addition, peripheral vascular disease/hypertension and cerebrovascular disease were the most common comorbidities (97; 26.6% and 63; 17.3%, respectively) and dementia was found in 7.7% (*N* = 28) of the tube‐fed patients. Most patients were at high risk of death (158; 43.3%), highly dependent on nursing care (129; 35.3%) and alert (214; 58.6%) on admission. However, the remaining 146 patients (40.0%) presented reduced levels of consciousness.

The main reason for inserting an NG/NET was decreased sensory state/level of consciousness/general state (132; 36.2%), and most tubes were placed blindly at the bedside of the patient (191; 52.3%). Epigastric auscultation was the clinical method most used by nurses to confirm feeding tube placement (67; 18.4%). In relation to the tube position, around 36% were positioned in the stomach and the mean length of NG/NET use was 14 days (8 ± 27.22). In addition, the mean LOS was 18 days (10.92; *SD*: 31.81), and of the 365 (100%) patients with NG/NET, 83 (22.7%) died (Files S2 and File S3).

There was an association between LOS and patient care complexity (*p* = .0179) (Table 1), age (*p* = .01) and length of NG/NET use (*p* = .01) (data not shown in table). No association was found between death and patient care complexity (*p* = .06578), disease severity (*p* = .5559) or level of consciousness (*p* = .06578) (Table 2). However, there was a statistically significant association between death and the patient's age (*p* = .0147) (data not shown in table).

**TABLE 1:** Analyses of association between length of stay and patient care complexity, disease severity and level of consciousness

Variables	Minimum	Median	Mean	Maximum	*SD*	Total	*p*‐value^a^
Patient care complexity							
Minimum	1.17	11.76	16.73	81.02	14.29	129	.0179
Intermediate	3.92	18.21	21.69	56.04	14.74	15	
High‐dependency	1.56	10.81	18.62	387.33	40.75	92	
Semi‐intensive	1.50	12.65	20.90	372.71	49.79	57	
Intensive	0.54	7.25	16.09	107.58	20.56	59	
Disease severity							
No risk	0.54	10.52	16.60	372.71	32.43	158	.0656
Low risk	1.52	10.40	17.48	107.58	17.83	73	
Moderate risk	1.50	10.96	15.31	54.69	12.36	101	
High risk	1.81	16.74	32.67	387.33	70.04	31	
Level of consciousness							
Alert	1.52	12.62	20.71	387.33	39.40	214	.1018
Confused	1.79	10.58	13.83	51.14	11.46	86	
Drowsy	1.69	13.29	12.58	33.46	8.13	28	
Unconscious	0.54	7.30	13.06	43.64	12.68	32	

Note

Abbreviation: *SD*, standard deviation.

^a^ Jonckheere–Terpstra test.

**TABLE 2:** Analyses of association between death and patient care complexity, disease severity and level of consciousness (*N* = 365)

Variables	Death						*p*‐value^a^
	Yes		No		Total		
	*N*	%	*N*	%	*N*	%	
Patient care complexity							
Minimum	32	8.8	85	23.3	117	32	.06578
Intermediate	6	1.6	8	2.2	14	3.8	
High‐dependency	19	5.2	70	1.9	89	24.4	
Semi‐intensive	7	1.9	46	12.6	53	14.5	
Intensive	17	4.7	34	9.3	51	14	
Total	81	22.2	243	66.6	324	88.8	
Disease severity							
No risk	39	10.7	108	29.6	147	40.3	.5559
Low risk	15	4.1	51	14	66	18.1	
Moderate risk	24	6.6	68	18.6	92	25.2	
High risk	5	1.4	22	6	27	7.4	
Total	83	22.7	249	68.2	332	91	
Level of consciousness							
Alert	41	11.2	156	42.7	197	54	.06578
Confused	25	6.8	52	14.2	77	21.1	
Drowsy	8	2.2	19	5.2	27	7.4	
Unconscious	9	2.5	19	5.2	28	7.7	
Total	83	22.7	246	67.4	329	90.1	

Note

Some frequencies do not sum to the total of 365 patients due to missing data.

^a^ Cochran–Armitage chi‐square.

The bivariate analysis shows a positive association between LOS and intensive care patients (*p* = .0133) and drowsiness (*p* = .0086) (Table 3). There was also a positive association between death and intensive care patients (*p* = .0294) (Table 4). The multivariate analysis showed statistical significance between LOS and intensive care (*p *= .0117) and drowsiness (*p* = .091) (Table 3). Statistical significance was also observed between death and high‐dependency care (OR: 2.52; 95% CI: 1.02–6.25; *p* = .0470), intensive care (OR: 6.20; 95% CI: 1.55–24.89; *p*=.0105) and semi‐intensive care (OR: 3.30; 95% CI: 1.22–8.91; *p* = .0194) (Table 4). When compared with patients receiving minimal care, patients highly dependent on nursing care, and semi‐intensive and intensive care presented, respectively, 2.52, 3.30 and 6.20 times greater likelihood of dying (Table 4).

**TABLE 3:** Factors associated with length of stay in patients with NG/NET

Variable	Bivariate analysis				Multivariate analysis			
	Estimate	Std. error	*t* value	Pr(>|t|)	Estimate	Std. error	*t* value	Pr(>|t|)
Intercept	2.4513	0.2565	9.5559	0.0000	2.3403	0.1253	18.6801	0.0000
Patient care complexity								
High‐dependency	0.1311	0.1564	0.8382	0.4026	0.1273	0.1536	0.8290	0.4078
Intensive	0.7640	0.3067	2.4914	0.0133	0.7733	0.3047	2.5382	0.0117
Intermediate	−0.0194	0.1589	−0.1221	0.9029	−0.0174	0.1545	−0.1123	0.9106
Semi‐intensive	−0.1588	0.1920	−0.8270	0.4089	−0.1387	0.1872	−0.7407	0.4595
Disease severity								
High risk	−0.1008	0.1515	−0.6651	0.5065	–	–	–	–
Moderate risk	−0.0128	0.1588	−0.0806	0.9359	–	–	–	–
No risk	0.2656	0.2187	1.2146	0.2255	–	–	–	–
Level of consciousness								
Confused	−0.2092	0.1226	−1.7064	0.0890	−0.2179	0.1235	−1.7648	0.0786
Unconscious	−0.0172	0.2173	−0.0792	0.9369	−0.0848	0.2149	−0.3947	0.6934
Drowsy	−0.5387	0.2037	−2.6441	0.0086	−0.5374	0.2047	−2.6251	0.0091
Age	−0.0014	0.0042	−0.3312	0.7408	–	–	–	–

**TABLE 4:** Factors associated with death in patients with NG/NET

Variable	Bivariate analysis				Multivariate analysis					95% CI	
	Estimate	Std. error	t value	Pr(>|t|)	Estimate	Std. error	t value	Pr(>|t|)	OR^a^	Lower	Higher
Intercept	−2.8718	0.8064	−3.5613	0.0004	−1.7519	0.4091	−4.2821	0.0000			
Patient care complexity											
High‐dependency	0.6819	0.4918	1.3865	0.1667	0.9243	0.4634	1.9945	0.0470	2.5202	1.0161	6.2506
Intensive	1.7853	0.8157	2.1888	0.0294	1.8253	0.7088	2.5750	0.0105	6.2044	1.5465	24.8925
Intermediate	0.2606	0.5126	0.5084	0.6116	0.4909	0.4917	0.9983	0.3189	1.6338	0.6232	4.2831
Semi‐intensive	0.9855	0.5681	1.7348	0.0838	1.1928	0.5075	2.3502	0.0194	3.2963	1.2190	8.9136
Disease severity											
High risk	−0.0660	0.4513	−0.1463	0.8838	–	–	–	–	–	–	–
Moderate risk	0.0031	0.4551	0.0068	0.9946	–	–	–	–	–	–	–
No risk	0.2981	0.6540	0.4558	0.6489	–	–	–	–	–	–	–
Level of consciousness											
Confused	0.3341	0.3356	0.9957	0.3202	–	–	–	–	–	–	–
Unconscious	0.0518	0.5581	0.0929	0.9261	–	–	–	–	–	–	–
Drowsy	−0.1689	0.5496	−0.3073	0.7589	–	–	–	–	–	–	–
Age	0.0193	0.0126	1.5280	0.1276	–	–	–	–	–	–	–
Time of NG/NET use	0.0013	0.0065	0.2050	0.8377	–	–	–	–	–	–	–

Note

Abbreviations: CI, confidence interval; NG/NET, nasogastric/nasoenteric tube; OR, odds ratio.

^a^ Adjusted by age, patient care complexity, disease severity, level of counsciousness and time of NG/NET tube use.

## 4 DISCUSSION

Most of the tube‐fed patients were male (52.6%), older adults (mean age of 64 years), at high risk of death (43.3%), with neoplasms (18.3%), diseases of the circulatory system (15.3%) and diseases of the nervous system (10.6%). Similar findings were identified in previous studies conducted with tube‐fed patients (Anziliero et al., 2017; Cervo et al., 2014; Ueno et al., 2018).

Acute and chronic illness, trauma and inflammation induce stress‐related catabolism increasing the risk of malnutrition (Lewis et al., 2018). In these cases, artificial feeding through a short‐term enteral access device is common in hospital settings. Although many outcome benefits can be observed in tube‐fed patients, adverse events may occur (Boullata et al., 2017) exposing patients to prolonged LOS and greater risk of death (Smith et al., 2018).

In this study, age was associated with LOS (*p* = .01) and death (*p* = .0147). With the number of older adults expected to rise significantly over the next few decades, changes to the healthcare system will need to take place to fulfil the needs of these people. Decreased lean body mass associated with medical comorbidities place older adults at high risk of developing frailty and malnutrition (Mundi et al., 2018). In these cases, an enteral access device may be required to provide adequate nutritional support (Volkert et al., 2006).

According to Sachdev et al. (2015), patients aged 60 years or over have a higher odds ratio for the need for enteral nutrition (4.188; *p* = .0019) compared with younger adults. However, hospitalized older adult women were the ones who most needed a nasogastric tube (NGT) compared with other enteral access devices (Ang et al., 2020; Sachdev et al., 2015).

The optimal tube location (gastric versus. small bowel) should be determined based on patient‐specific factors (i.e., patient's disease state, gastrointestinal anatomy, gastric and intestinal motility and function and expected duration of enteral nutrition) (Boullata et al., 2017). In general, gastric access is the first option and is appropriate for patients with a functional stomach, free of delayed gastric emptying, obstruction or fistula. Clinicians may prefer small bowel feeding for patients with gastric outlet obstruction, severe gastroparesis and for those who are at high risk of aspiration (Boullata et al., 2017; Lord, 2018). This includes patients with decreased level of consciousness, diminished cough or gag reflex, impaired lower oesophageal sphincter, neurologic deficits, severe gastroesophageal reflux disease, severe gastroparesis, elevated gastric residual volumes and emesis (Lord, 2018). Patients who need simultaneous gastric decompression with small bowel feedings can be best accommodated using a dual‐lumen tube (Boullata et al., 2017; Lord, 2018).

In the present study, 7.7% of the tube‐fed patients had dementia recorded in their medical chart. Although enteral nutrition is always the first choice for patients with swallowing limitations and intact bowel function (Volkert et al., 2006), it is not recommended for older adults, especially those with advanced dementia (American Geriatrics Society Ethics Committee and Clinical Practice and Models of Care, C, 2014). In these cases, careful hand feeding should be provided as this has been shown to be as good as tube feeding considering the outcomes of death, aspiration pneumonia, functional status and comfort (American Geriatrics Society Ethics Committee and Clinical Practice and Models of Care, C, 2014). Therefore, nurses and the healthcare team should carefully consider the administration and choice of enteral nutrition methods, taking into account the prognosis of the patients (Hayashi et al., 2020).

In the present study, LOS was associated with patient care complexity (*p* = .0179) and most patients required the insertion of an NG/NET due to decreased level of consciousness. The association between LOS and care complexity in tube‐fed patients can be explained by the fact that enteral feeding is the method of choice for the nutritional support of critically ill patients (Seron‐Arbeloa et al., 2013; Silva et al., 2018), as these patients usually present altered metabolism and varying needs during the different phases of illness (Boullata et al., 2017).

In a study that analysed adverse events in critically ill patients from a general intensive care unit of a private Brazilian hospital, the results showed that patients who presented the event had longer LOS, greater mean age, higher scores on the APACHE II scale, higher risk on the Braden scale and lower scores on the Glasgow Coma Scale (Ortega et al., 2017).

Nurses need to consider the best choice of enteral access device and where the tip should be placed, as well as recommendations for changes in the patient's care plan (McGinnis et al., 2010). There are principles (Boullata et al., 2017; Lord, 2018; McGinnis et al., 2010) to help guide nursing practice; however, decisions should also be patient‐specific (Boullata et al., 2017; McGinnis et al., 2010). Furthermore, a multidisciplinary team of competent clinicians working in partnership is required to provide safe nutrition care for tube‐fed patients (Hudson & Boullata, 2014). Optimal communication among all members of the multidisciplinary team and standardization across all steps of the enteral nutrition process are an important risk management strategy to reduce NG/NET‐related complications and to improve patient outcomes (Boullata et al., 2017; Hudson & Boullata, 2014).

The LOS was associated with length of NG/NET use (*p* = .01). According to the ASPEN safe practices for enteral nutrition therapy, a nasoenteric or oroenteric tube should be fitted in patients who require enteral nutrition for up to approximately 4–6 weeks (Boullata et al., 2017; Lord, 2018). In the present study, the tube‐fed patients presented a mean LOS of 18 days and a mean length of NG/NET use of 14 days, which may explain the result found. According to organizational policies and procedures, frequent monitoring and continual review of the indications for continued use of any NG/NET are necessary, including the consideration of changes to the care goals (Boullata et al., 2017; Prabhakaran et al., 2012).

In Brazil, the nursing team plays an increasingly pivotal and active role in enteral nutrition therapy. The nursing team is supervised by the Registered Nurse, who is directly responsible for the selection, standardization, purchasing and acquisition of the equipment and materials used for the administration of enteral nutrition, as well as for the processes of administration, prescription of nursing care at the hospital, outpatient and home level and patient and family guidance on the safe handling of enteral access devices and enteral nutrition. Accordingly, nurses are an integral part of the multidisciplinary nutritional therapy team, together with at least one professional of each category, namely physician, dietician and pharmacist, a team preferably composed of specialists in the area related to nutritional therapy (Brasil, 2000). Other important points refer to the documentation of the size and manufacture/model of the tube once it is fitted (Boullata et al., 2017), the incremental marking on the tube at the exit site (nares, mouth or stoma) (Lord, 2018) and the course of the tube and location of its tip (Boullata et al., 2017; Lord, 2018).

Previous research has highlighted the important role played by the nursing team in the prevention of adverse events related to enteral nutrition. The results showed that these professionals were able to recognize potential risks and intervene early, contributing to the maintenance of life (Carrasco et al., 2018). However, flaws in nursing care are still found due to theoretical limitations and/or negligence (Medeiros et al., 2015), with divergence between what is practiced and what is recommended in national (Brasil, 2000) and international (Boullata et al., 2017) enteral nutrition guidelines.

Of the 365 tube‐fed patients in this study, 22.7% died, with this result being lower when compared with a study conducted in another Brazilian general hospital (26.4%) (Ueno et al., 2018). This difference could be explained by the differences in the study population. While we evaluated data from tube‐fed patients admitted to clinical wards, Ueno et al. (2018) included all patients with enteral nutrition therapy at the hospital sites of their study.

In the present study, death was not associated with patient care complexity (*p* = .06578), disease severity (*p* = .5559) or level of consciousness (*p* = .06578). These results corroborate those of studies conducted with patients with acute stroke. According to the researchers, the early use of an NGT in intensive care units did not increase the mortality or poor functional outcomes, concluding that this medical device can be safely used with these patients (Kalra et al., 2016; Vest et al., 2018). In addition, the NGT is the recommended route for early nutrition in patients with reduced levels of consciousness. Early nutrition is essential for the caloric–protein intake in these patients, and nurses can perform this procedure safely and efficiently (Singer et al., 2019). Another study that investigated the results of older adult patients with NGT and parenteral nutrition found that these patients had a higher risk of death, more complications, prolonged LOS and less chance of being discharged to home when compared with patients with a short‐term enteral access device (Honda et al., 2020).

In contrast to these results, a study developed in nine institutions in Europe and Australia showed that the use of an NGT was associated with a higher risk of death. However, the result was limited to more medically fragile patients, suggesting the importance of a prognostic evaluation for the insertion of the tube, especially in older adult patients (Veronese et al., 2020). Another study carried out in Singapore compared the care needs of patients with NGT with those of patients with percutaneous endoscopic gastrostomy (PEG). The results showed that patients with a short‐term enteral access device had greater care needs, with a significant difference in the final CCI score (*p* = .002). In addition, the patients with NGT developed a significantly higher number of mechanical complications than the patients with PEG (Ang et al., 2020). It can be presumed that several factors are associated with death in tube‐fed patients and nurses should be aware of these risks to improve patient safety.

In the multivariate analysis, statistical significance was observed between the LOS and intensive care (*p* = .0117) and drowsiness (*p* = .091). Statistical significance was also observed between death and high‐dependency (*p* = .0470), intensive (*p* = .0105) and semi‐intensive (*p* = .0194) care. In addition, patients highly dependent on nursing care, and semi‐intensive and intensive care had a greater likelihood of dying when compared with patients receiving minimal care. The LOS was also associated with advanced age, disease severity, adverse events and death in previous studies (Daud‐Gallotti et al., 2012; Oliveira et al., 2016; Ortega et al., 2017; Roque et al., 2016; Soares et al., 2015).

Tube‐fed patients require continuous monitoring and competent nursing care to improve patient outcomes. It should be emphasized that patients with more severe diseases require more nursing care hours per patient, resulting in increased level of nursing workload, patient safety incidents (Agency for Healthcare Research and Quality, 2019; Cho et al., 2003; Kang et al., 2016; Oliveira et al., 2016), morbidity and mortality (Agency for Healthcare Research and Quality, 2019), and increased medical costs (Cho et al., 2003). Furthermore, missed nursing care is relatively common on inpatient wards and is strongly associated with a higher number of patients per nurse (Agency for Healthcare Research and Quality, 2019). Nurses' vigilance at the bedside is essential to ensure patient safety and the quality of care (Agency for Healthcare Research and Quality, 2019). Therefore, appropriate nurse staffing should be considered a patient safety issue, as nurse staffing ratios have an impact on patient safety.

In a previous study conducted in Ontario complex continuing care hospitals, the risk factors for prolonged LOS were functional and cognitive impairment, greater pressure ulcer risk, paralysis, antibiotic resistance and HIV infection, need for a feeding tube, dialysis, tracheostomy, ventilator or a respirator use and psychological therapy (Turcotte et al., 2019). The present results indicate the relevance of evidence‐based practices and cost‐effectiveness studies in addressing patients with NG/NET, especially older adult patients, with greater disease severity, higher care complexity and prolonged LOS.

According to a study conducted with adult, critically ill patients (Cangelosi et al., 2011), compared with parenteral nutrition, enteral nutrition reduced the risk of potentially life‐threatening serious infections and the risk of potentially life‐threatening non‐infectious events and suggested a reduction in mortality, although this result was not statistical significant. Enteral nutrition also reduced the LOS, length of time in the ICU and duration of nutritional treatment.

Early enteral nutrition for critically ill patients has been recognized as an important factor to improve patient survival and significantly reduce total treatment costs (Doig et al., 2013). However, in a literature review, which included 25 studies with 8,816 participants, the results showed insufficient evidence to determine whether enteral nutrition is better or worse than parenteral nutrition, or enteral nutrition and parenteral nutrition combined, in reducing hospital mortality, at 30, 90 and 180 days, the number of days without ventilation and the occurrence of adverse events (Lewis et al., 2018).

The ASPEN confirmed the benefits of early enteral nutrition for critically ill patients, if oral intake is not possible, through clear evidence to support change in care practice. Furthermore, early enteral nutrition (within 48 hr) should be initiated instead of early parenteral nutrition, considering the risk of malnutrition of adult patients in a serious condition (Singer et al., 2019). It should be emphasized that variables related to LOS and death are dependent variables that vary due to the severity of the disease and the therapeutic requirements; therefore, different results can be observed among studies.

To maximize the benefits of enteral nutrition through a NG/NET while minimizing adverse events requires a systematic care approach to be in place. This includes open communication, standardization and incorporation of best practices into the enteral nutrition process (Boullata et al., 2017).

This is the first large‐scale study in Brazil and Latin America documenting the factors affecting LOS and death in tube‐fed patients. The results provide valuable information for care planners and health system administrators working to improve the safety of these patients in acute care settings.

### 4.1 Limitations

There are some limitations to be addressed in this study. In particular, there was no comparator non‐NG/NET group of patients. Considering the cross‐sectional nature of the data, it is not possible to determine the direction of key relationships due to the existence of multiple confounding variables. In addition, the accuracy of the clinical data depended on the quality of the medical records. Prospective studies are needed to understand the predictors of LOS and death in patients with short‐term enteral access devices.

## 5 CONCLUSION

In the present study, most patients were male, older adults, with high risk of death and highly dependent on nursing care. The LOS was associated with age, patient care complexity and length of NG/NET use, while death was only associated with patient age. In the multivariate analysis, patients highly dependent on nursing care, and intensive and semi‐intensive care had a greater likelihood of dying when compared with patients receiving minimal care. Screening for factors affecting LOS and death in tube‐fed patients is important to plan effective nursing care and to improve patient outcomes.

## 6 RELEVANCE FOR CLINICAL PRACTICE

The results may help nurses and other members of the multidisciplinary team to improve institutional policies and procedures related to NG/NET use. Based on the results, certain objective assessments should be investigated during the hospital stay, especially in older adults with a short‐term enteral access device. Clinical vigilance and patient safety protocols may help nurses to improve patient outcomes and the quality of care provided to tube‐fed patients.

## CONFLICT OF INTEREST

The authors have no conflicts of interest concerning this study.

## Supporting information

File S1

File S2

File S3

## Data Availability

The data that support the findings of this study are available in the supplementary material of this article. The authors are aware of and authorize the availability/accessibility of data.
