# Supplementary material for: Factors associated with length of stay and death in tube‐fed patients: A cross‐sectional multicentre study
Source: Nurs Open. 2021 Jan 27;8(5):2509–19. doi: 10.1002/nop2.774 (PMC8363365; doi:10.1002/nop2.774)
Supplement: Supplementary file 2 — File S2 [file NOP2-8-2509-s001.docx]

**Table** Characteristics of patients (N=365)

| **Variables** | **n** | **%** |
| --- | --- | --- |
| ***Sex*** |  |  |
| Male | 192 | 52.6 |
| Female | 171 | 46.8 |
| ***Race*** |  |  |
| White | 240 | 65.8 |
| Black | 64 | 17.5 |
| Other | 47 | 12.9 |
| ***Marital status*** |  |  |
| Married | 161 | 44.1 |
| Widowed | 74 | 20.3 |
| Single | 65 | 17.8 |
| Separated/divorced | 38 | 10.4 |
| Cohabiting | 13 | 3.6 |
| Other | 3 | 0.8 |
| ***Educational level*** |  |  |
| Early childhood education | 131 | 35.9 |
| Primary education | 74 | 20.3 |
| Secondary education | 46 | 12.6 |
| Higher education | 18 | 4.9 |
| Without studies | 76 | 20.8 |
| ***State*** |  |  |
| São Paulo | 231 | 63.3 |
| Acre | 65 | 17.8 |
| Rio Grande do Sul | 31 | 8.5 |
| Minas Gerais | 22 | 6.0 |
| Ceará | 13 | 3.6 |
| Other | 2 | 0.6 |
| ***Period of admission to the ward*** |  |  |
| Afternoon | 181 | 49.6 |
| Morning | 97 | 26.6 |
| Night | 81 | 22.2 |
| ***Using an NG/NET on admission*** |  |  |
| Yes | 214 | 58.6 |
| No | 151 | 41.4 |
| ***Medical discipline*** |  |  |
| General clinical | 144 | 39.5 |
| Oncology | 51 | 14.0 |
| Neurology | 33 | 9.0 |
| Geriatrics | 31 | 8.5 |
| Gastroenterology | 27 | 7.4 |
| Infectious diseases | 12 | 3.3 |
| Cardiology | 10 | 2.7 |
| Nephrology | 11 | 3.0 |
| Other | 46 | 12.6 |

Note: Some frequencies do not sum to the total of 365 patients due to missing data

Abbreviations: NG/NET, nasogastric/nasoenteric tube
