# Supplementary material for: Factors associated with length of stay and death in tube‐fed patients: A cross‐sectional multicentre study
Source: Nurs Open. 2021 Jan 27;8(5):2509–19. doi: 10.1002/nop2.774 (PMC8363365; doi:10.1002/nop2.774)
Supplement: Supplementary file 3 — File S3 [file NOP2-8-2509-s003.docx]

**Table.** Clinical and therapeutical variables of the study participants (N=365)

| **Variables** | **n** | **%** |
| --- | --- | --- |
| ***The five-primary reason for hospital admission-ICD-10*** |  |  |
| Neoplasms [tumors] (C00-D48) | 55 | 18.3 |
| Diseases of the circulatory system (I00-I99) | 46 | 15.3 |
| Diseases of the nervous system (G00-G99) | 32 | 10.6 |
| Diseases of the respiratory system (J00-J99) | 31 | 10.3 |
| Diseases of the digestive system (K00-K93) | 25 | 8.3 |
| ***The five-primary comorbidities*** |  |  |
| Peripheral vascular disease - hypertension | 97 | 26.6 |
| Cerebrovascular disease | 63 | 17.3 |
| Uncomplicated diabetes | 46 | 12.6 |
| Malignant tumor or metastasis | 41 | 11.2 |
| Severe or moderate kidney disease | 38 | 10.4 |
| ***Patient Classification System*** |  |  |
| High dependency care | 129 | 35.3 |
| Intermediate care | 92 | 25.2 |
| Semi-intensive care | 59 | 16.2 |
| Minimal care | 57 | 15.6 |
| Intensive care | 15 | 4.1 |
| Not applicable | 8 | 2.2 |
| ***Disease severity*** |  |  |
| High risk | 158 | 43.3 |
| Moderate risk | 100 | 27.4 |
| Low risk | 71 | 19.5 |
| No risk | 31 | 8.5 |
| ***Level of Consciousness*** |  |  |
| Alert | 214 | 58.6 |
| Confused | 86 | 23.6 |
| Response to pain | 32 | 8.8 |
| Unconscious | 28 | 7.7 |
| ***Reason for using the tube*** |  |  |
| Decreased sensory state/level of consciousness/general state | 132 | 36.2 |
| Dysphagia | 97 | 26.6 |
| Inappetence/low oral acceptance | 62 | 17.0 |
| Malnutrition | 27 | 7.4 |
| Use of orotracheal tube | 8 | 2.2 |
| ***Method used to insert the tube*** |  |  |
| Blind / bedside | 191 | 52.3 |
| Not applicable | 141 | 38.6 |
| Other | 15 | 4.1 |
| Endoscopy | 10 | 2.7 |
| ***Position of the distal tip*** |  |  |
| Gastric | 131 | 35.9 |
| Enteric (duodenum/jejunum) | 129 | 35.3 |
| Not applicable | 99 | 27.1 |
| ***Method used to confirm the tube placement*** |  |  |
| Not applicable | 144 | 39.5 |
| Epigastric auscultation only | 67 | 18.4 |

| Epigastric auscultation and radiological examination (X-ray) | 50 | 13.7 |
| --- | --- | --- |
| Radiological examination only (X-ray) | 44 | 12.1 |
| Epigastric auscultation, aspiration of gastric contents and  radiological examination (X-ray) | 20 | 5.5 |
| Epigastric auscultation and aspiration of gastric contents | 19 | 5.2 |
| Other | 8 | 2.2 |
| Only aspiration of gastric contents | 3 | 0.8 |

Note: Patients could have more than one comorbidity. The option “Not applicable” was selected for the patients admitted to the clinical ward with the NT/NET.

Abbreviations: ICD-10, International Classification of Diseases, 10th revision.
